# Next of kin’s experiences of registered nurses’ leadership close to older adults in municipal home care in Sweden: an interview study

**DOI:** 10.1186/s12912-021-00745-6

**Published:** 2021-10-29

**Authors:** Maria Claesson, Lise-Lotte Jonasson, Karin Josefsson

**Affiliations:** 1 Faculty of Caring Science, Work Life and Social Welfare, University of Borås, 501 90 Borås, Sweden; 2 Department of Nursing School of Health and Welfare, Jönköping University, Jönköping, Sweden; 3 Faculty of Health, Science and Technology, Department of Health Sciences, Karlstad University, Karlstad, Sweden

**Keywords:** Next of kin, Registered nurse, Leadership, Older adult, Municipal home care, Interviews

## Abstract

**Background:**

Next of kin to older adults over 65 years in municipal home care are concerned whether their older adults’ needs are being met. In municipal home care, the registered nurses’ leadership is important and complex, entailing multi-artist skills involving the older adults and their next of kin. Yet, little is known about next of kin’s experiences of registered nurses’ leadership. Thus, the aim of this study was to explore next of kin’s experiences of registered nurses’ leadership close to older adults in municipal home care.

**Methods:**

Individual telephone interviews were conducted with next of kin (*n* = 11) of older adults from April to September 2020 in two municipalities in western Sweden. Data were analysed using qualitative content analysis.

**Results:**

The results are presented with the theme, registered nurses do what they can, including two categories, interaction and competence, and the subcategories, relationship, communication, availability, responsibility, team leadership and cooperation. Registered nurses’ leadership was experienced as a balancing act between their commitments and what they were able to achieve.

**Conclusions:**

Next of kin’s experiences of registered nurses’ leadership can contribute knowledge that will strengthen and prepare registered nurses for their leadership roles. This knowledge can support the development of policies for organisational preconditions that ensure quality and safe care to older adults in municipal home care.

## Background

Being the next of kin to older adults in need of municipal home care often entails concern regarding whether their older adults’ needs are being met and whether the healthcare providers are being respectful towards their loved ones [1]. Therefore, next of kin wish to be included in the healthcare teams surrounding their older adults [2] and to cooperate with the care professionals at different levels in the healthcare system [3].

Close involvement in their older adults’ municipal home care [1] can be a heavy burden on the next of kin’s lives, giving rise to contradictory emotions and implicit requirements both from the older adults and society [4]. Next of kin sometimes also have to cope with excessive responsibilities related to care [5] to ensure that the older adults receive the care to which they are entitled [6].

It is important for the next of kin to feel like they can be involved in the care of their loved ones, without the responsibility of the primary caregiver [7]. Nevertheless, it is not always clear who is responsible for the municipal home care and what the different roles of the team members are [8]. For example, next of kin do not always know the difference between registered nurses’ (RNs’) responsibilities for home care and the responsibility of the professionals regarding social services. These ambiguities in who is responsible for what can also contribute to next of kin not knowing to whom to turn when problems arise [3, 9].

RN leadership is founded in the caring sciences; thus, RNs must possess a clear patient perspective [10], which is important for older adults in municipal home care in order to maintain good and safe care [11–13]. In Sweden, municipal home care and social services are included in the welfare system, and older adults living in their home are legally entitled to assistance from the municipalities to meet older adults’ individual needs. Home care in Sweden is provided in the older adults’ homes, consistently over time [14].

Leadership is a part of RNs’ professional role [15]’ RNs’ leadership close to older adults in municipal home care requires the RNs be working independently as well as together with other healthcare professionals to support older adults’ care needs [11, 16]. Further, the requirement to be an RN in municipal home care in Sweden is at least a three-year education program with a bachelor’s degree but preferable is a master’s degree in specialists nursing education [17].

RNs have a personal responsibility to perform leadership [12] and working in older adults’ homes places demands on RN flexibility and competence [16, 18].

Previous research [11] concluded that RN leadership close to older adults in municipal home care requires the RNs to be multi-artists, i.e. the RNs are responsible for collaboration at the interpersonal level, which involves teamwork among the RNs, other professionals, older adults and next of kin. Further, RNs have to be able to engage in and be sensitive to older adults’ individual needs and their whole beings [11], as well as to provide support to both the older adult and their next of kin [3, 15]. RNs have an ethical responsibility for this leadership [12, 19], which involves interacting with older adults and their next of kin as partners in their care.

Since research shows that next of kin’s involvement and RN leadership are important for older adults in municipal home care, there is a need to better understand how next of kin experience RN leadership. Understanding next of kin’s experiences is crucial to the development of RN leadership and to enhance municipal home care for older adults. Therefore, this study aimed to explore next of kin’s experiences of RN leadership close to older adults in municipal home care.

## Methods

### Design

Qualitative interviews were conducted from April to September 2020 in two municipalities in western Sweden. Individual telephone interviews [20] were conducted with next of kin (*n* = 11) of older adults in municipal home care. An explorative and inductive design was used [21]. Data were analysed by qualitative content analysis [22–24].

### Inclusion and exclusion criteria

The inclusion criteria were the next of kin of older adults with at least 1 year’s experience in municipal home care and the ability to participate in a telephone interview. The exclusion criteria were cognitive impairment and the need for an interpreter during the telephone interview: the next of kin had to be able to conduct the interview in Swedish.

### Participants and procedure

After gaining permission from municipal home care managers in two municipalities, one municipality in a city area and one municipality in a rural area, the member of the research group who carried out the interviews contacted an RN to get in touch with next of kin of older adults who met the inclusion criteria. The participants were selected through purposive sampling [21]: they all had experiences of RN leadership close to older adults in municipal home care. Eight next of kin (*n* = 8) provided their contact details via the RN. Contact details to three participants (*n* = 3) were obtained via next of kin who did not meet the inclusion criteria themselves but were acquainted with individuals who did. In total, 11 next of kin (*n* = 11), including women (*n* = 10) and a man (*n* = 1), chose to participate and received information by letter about the study. The next of kin had different relationships with their older adults (Table 1). The next of kin’s mean age was 70 years (min–max = 47–83 years) (Table 1), and they had 1–10 years’ experience with RN leadership in municipal home care.

**Table 1:** Description of participants (*n* = 11)

Next of kin	Relationship	Age	Gender	Experience of registered nurses’ leadership close to older adults in municipal home care
1	Wife	75	Female	Three years
2	Wife	83	Female	Five years
3	Wife	70	Female	One year
4	Wife	70	Female	One year
5	Husband	67	Male	One year
6	Partner	75	Female	Ten years
7	Sister	74	Female	Five years
8	Daughter	68	Female	Six years
9	Daughter	65	Female	One year
10	Daughter-in-law	48	Female	One year
11	Daughter-in-law	47	Female	One year

### Data collection

An interview guide was created through literature studies and in discussions with the members of the research team for the purpose of achieving trustworthiness [22–24]. The interview questions were constructed to capture different experiences of the phenomenon with the approach of ‘open questions’ to improve the dependability and credibility of the findings [25, 26]. The next of kin were contacted by a member of the research group to book an appointment for a telephone interview. The next of kin chose the time to be interviewed. Before the start of the interview, the interviewer briefly presented the aim of the study to the next of kin. The interview then started with two open questions: (1) ‘Can you tell me how you experience the contact that you have with the RN who visits your older adult at home?’ and (2) ‘Can you tell me how you experienced the RN’s leadership close to the older adult in municipal home care?’ Two subsequent questions were then posed: ‘Is there something you miss in your contact with the RN?’ and ‘Would you want something to be different in your contact with the RN?’ Follow-up questions were asked when needed. Examples included ‘How?’, ‘What does that mean?’, ‘Can you give some examples?’ and ‘Is there any particular situation of which you are thinking?’ All the interviews ended with the question: ‘Is there anything you want to add before the end of the interview?’ The interviews lasted an average of 37 min (min–max = 19–79 min) and were recorded on a tape recorder.

### Data analyses

The data were analysed using qualitative content analysis [22–24]. The interviews were transcribed verbatim and read several times by one member of the research team to get an overview of the content. The transcripts were then sorted into meaning units which were condensed and abstracted into codes. The codes were compared to each other, focussing on variations of differences and similarities, which led to the determination of a theme with two underlining categories and six subcategories (Table 2). The categories and subcategories were then discussed at several research group meetings. This analysis proceeded by moving back and forth between the whole text and parts of the text until consensus was reached.

**Table 2:** Next of kin’s experiences of registered nurses’ leadership close to older adults in municipal home care

Theme	
Registered nurses do what they can	
**Category**	**Subcategory**
Interaction	Relationship
	Communication
	Availability
Competence	Responsibility
	Team leadership
	Cooperation

## Results

The results describe next of kin’s experiences of RN leadership close to older adults in municipal home care with the theme, registered nurses do what they can, including two categories, interaction and competence, and six subcategories (Table 2).

### Registered nurses do what they can

The next of kin to older adults in municipal home care experienced that the RNs managed their leadership in the best way considering their circumstances. The RNs managed to lead despite a lack of organisational preconditions, such as distance collaboration or other healthcare professionals to help organise. The next of kin experienced RN leadership as a balancing act between the RNs’ commitments and what they were actually able to accomplish in their leadership, such as being an adequate leader to the nursing assistants without influencing their working conditions. Moreover, this leadership was extended to include interactions between the RNs, the next of kin and the older adult, which consisted of relationships, communication and availability. The next of kin also experienced RN leadership to include competence in the RNs’ professional responsibilities, being a team leader and cooperation. Next of kin who participated illustrated RN leadership as

‘ *… the registered nurses had no organisational preconditions, but there is no shadow over them. The registered nurse is super, but I see that the organisation has not arranged it in that way...*’ [9]

#### Interaction

##### Relationship

Next of kin experienced RN leadership as including a relationship. The relationship was experienced to be mutual but also to include trust, respect and safety between the RN, the next of kin and the older adult. Having a relationship with the RN created satisfaction for the next of kin; however, there were next of kin who experienced a lack of relationship with the RNs because they had never met the RN in person. This relationship also included the next of kin experiences that the RNs were taking an ethical approach towards the older adult: the RN did not talk over the head of the older adult to the next of kin; instead, the RN spoke directly to the older adult in care. The experienced relationship included reliability, which meant the next of kin felt they could trust the RNs. Nonetheless, there were also experiences of a lack of trust when the RNs did not keep their promises.

‘ *… I trust the registered nurse and my older adult trusts the registered nurse. The relationship is mutual, as I think it should be...*’ [8]

Feeling safe was experienced in the relationship with RN leadership, both for the next of kin’s older adult and for the next of kin themselves. Safety was experienced, for example, when the next of kin knew they could phone the RN if they needed information or if they were worried about things related to their older adult. In contrast, next of kin experienced a lack of safety when unknown healthcare workers entered their homes but after they got to know each other, it was no longer a problem. The next of kin also experienced safety in the relationship when the RN contacted them to ask about things the older adult could not always answer for themselves. In this way, the next of kin experienced safety because they experienced the RNs’ concerns about the relationship with the next of kin and their older adult.

‘ *… but I must say that I am satisfied with the registered nurse in home care. I feel safe and ... that safety is important...*’ [6]

‘ *… especially in the beginning, it felt good when the registered nurse wanted to find out the background facts. It felt safe that it was important to the registered nurse that [the care] should be as good as possible....*’ [7]

##### Communication

Communication was experienced when the RNs provided information to the next of kin about their older adults’ treatment and expected effects. Communication also included when next of kin experienced there was close dialogue between the RNs and the home care assistants, which was important for the care of the older adult. Further, communication included when the next of kin had a telephone number, so they could call the RN directly when they needed. Nevertheless, not all next of kin had direct phone numbers to the RNs; this caused anxiety for the next of kin who had to call many different numbers to get in touch with their RN, and this was experienced as a lack of communication in their RN’s leadership.

*‘ … I do not call the registered nurse to say that my husband has slept badly. It is the home care assistant who takes care of that...*’ [1]

*‘ … I have 15 different phone numbers here, but they do not answer...*’ [8]

##### Availability

The next of kin experienced availability when they could easily contact an RN who would then visit the older adult’s home right away. There were also next of kin who wished they had a scheduled time to telephone their RN because they felt the RN was usually busy and difficult to reach, and they did not want to disturb the RN unnecessarily. RN availability was also experienced when an RN made time to converse with the next of kin or the older adult. However, occasionally, the next of kin wished that the RN was available more often even if there was no special issue to discuss. Availability was also experienced when the next of kin had contact with the same RN over time, i.e. when the older adult had their own RN. In general, the older adults were satisfied when another RN visited—for instance, when the regular RN was on vacation—but this could also create anxiety for the next of kin. The next of kin experienced that availability implied regularity, which occurred when the same RN visited regularly according to a schedule.

*‘ … I have a telephone number that goes directly to the registered nurse; usually, it is our registered nurse who answers. If my husband has a fever or something, the registered nurse always comes here the same day...*’ [5]

‘*...it is the same registered nurse who comes to visit every Monday morning*...’ [2]

#### Competence

##### Responsibility

The next of kin experienced the RN as having overall responsibility for the home care to the older adult, even though it was not always the RNs themselves who performed the care. They experienced that there were ambiguities in the RNs’ leadership responsibilities when it came to homecare assistants who performed the care. Responsibility was also experienced as RNs working according to routines to maintain patient safety. The next of kin experienced care for their older adult as working well when routines were followed. They also experienced responsibility in the RNs’ leadership as including the RNs having professional skills and performing their duties in an exemplary manner.

‘*...the registered nurse has overall responsibility and control of the situation, so there will be nothing wrong with medication and other things*...’ [4]

Further RN leadership roles were experienced by providing nursing interventions. Nursing interventions included blood sampling, drug administration, wound dressing, assessments, blood pressure check-ups, prescription of various aids and all activities that the next of kin linked to the RNs’ professional roles. Nonetheless, next of kin also experienced shortcomings in RN leadership towards carrying out their nursing intervention responsibilities: these shortcomings were primarily experienced when home care assistants did not complete their assignments following current guidelines. For example, the home care assistants did not always inform the RN when drugs or medical supplies were nearly expended and needed to be ordered. This caused concern for the next of kin who had to solve the problem on their own.

‘*...the home care assistants did not want to give my mother her medication, even if they were supposed to do that. Then, you had to have that discussion with the registered nurse. I thought my mother’s medication was very badly taken care of when she was in so much pain. It did not work at all*...’ [11]

##### Team leadership

Next of kin experienced RN leadership to include being a team leader. Being a team leader was experienced when the RNs introduced new colleagues and home care assistants to their work duties and also involved the next of kin and the older adult in the care. Further, team leadership included delegating nursing interventions to home care assistants. The next of kin also experienced ambiguities in the RNs’ abilities to lead home care assistants and related this to RNs sometimes lacking the organisational preconditions necessary to be team leaders. Next of kin associated these difficulties with the fact that the RNs were not the home care assistants’ managers; therefore, the RNs may have lacked a clear mandate as to whether they should act as team leaders for the home care assistants.

*‘ … since the registered nurse is not the manager for the home care assistants, the registered nurse feels that they cannot do anything...*’ [8]

##### Cooperation

The next of kin experienced RN leadership as including cooperation, which could involve cooperation between RNs and physicians in both primary and specialist care. Cooperation was also experienced when the RN was the one who coordinated and adapted nursing interventions for the older adult when several care providers were involved, such as a physiotherapist and an occupational therapist. The next of kin experienced that the RNs’ leadership entailed difficulties in cooperation between home care and primary care. Since the contact between the RN and the physician in primary care was usually carried out at a distance, there were also difficulties related to there being a lack of physician to contact when needed. The next of kin experienced disappointment when cooperation was carried out at a distance and when the physicians were unavailable, but they also understood that it was a situation that the RNs could not influence.

*‘...after all, it is the registered nurse who has knowledge about the older adult’s care needs and conveys it to the physicians...’* [10].

‘*...the registered nurse also has a hard time because they only get the opportunity to meet the physician at the healthcare centre on Wednesdays...*’ [3]

## Discussion

The aim of this study was to explore next of kin’s experiences of RN leadership close to older adults in municipal home care. The results showed that next of kin experienced RN leadership as a balancing act where the RNs did what they could to satisfy the older adults’ individual care needs without controlling the organisational resources required for the performance of the care. Despite a lack of organisational preconditions, such as distance collaboration, next of kin experienced the RNs’ leadership as including an interaction between the RN, the next of kin and the older adult, consisting of relationship, communication and availability. Our results also showed that RN leadership included being competent, completing their professional responsibilities, being a team leader and cooperating.

Since RN leadership implies a complex and challenging role, RNs may find it difficult to make demands on the organisation in order to have the preconditions required to be able to perform their leadership at the highest level. Strandås, Wackerhausen and Bondas [27] demonstrated that the RNs in home care, despite a lack of organisational preconditions, prioritised the close relationship with their patients and their next of kin. Related to results in this study, O’Rourke [28] also argued for the importance of developing strategies to create a dedicated professional nurse workforce, since there is a link between professional role clarity and work engagement. Further, recent research [29] showed that managers had limited knowledge of expectations and perceptions regarding RNs’ leadership roles, which might be one reason why RNs lack preconditions to perform their leadership. Södersved Källestedt, Asp, Letterstål and Widarsson [30] confirmed that the RNs themselves were not always prepared for their leadership role; thus, they lacked the leadership skills. Earlier research [31] concluded there is a need to develop programmes that allow RNs to become confident in their roles as leaders.

The results also revealed that there were next of kin who experienced RNs they could only contact by telephone, but they had an unspoken desire to meet the RN in person. Since next of kin want to be involved in the healthcare process of their older adults, it could be assumed that it was challenging for next of kin to have a relationship with the RNs without personal contact. In caring sciences that take a patient perspective approach [32], RN leadership includes maintaining a relationship between the older adults and their next of kin, and this relationship is crucial to the level of care provided. Wälivaara, Sävenstedt and Axelsson [33] confirmed that a trusting relationship was needed to provide good nursing care at home. Peter and Morgan [34] also established that trust is a moral dimension of a relationship and is important in every nursing relationship.

Our results showed that next of kin included RNs’ ethical approach towards their older adults when the RN talked directly to the older adult instead of through the next of kin. Similar research [35] showed that RNs had to deal with ethical challenges related to next of kin, and those challenges were different depending on the next of kin’s own personality. Jonasson, Liss, Westerlind and Berterö [36] also showed that RNs’ ethical values were presented through respectful and caring engagements in the meeting with the older adult and their next of kin, which our results corroborate.

Next of kin experienced that RNs mostly had a close dialogue with the home care assistants and that it was easy to call the RNs when needed. Occasionally, there were next of kin who had to call many different phone numbers to get in touch with their RNs, which was experienced as unsatisfactory for the next of kin and their older adults. This result is worrying since it makes it difficult for the next of kin to participate in the care of their older adult, and this lack of communication also contributes to next of kin having to take responsibility for some aspects of care that they do not want. Current research [37] also concluded that RN leadership roles are important in the design and implementation of care models for older adults and in support of their next of kin.

The results indicated that next of kin experienced RN leadership as consisting of having an overall responsibility for satisfying their older adults’ care needs based on routines and guidelines. However, next of kin also experienced inconsistencies in those responsibilities related to the fact that there were nursing assistants who were providing the care most of the time. These findings are similar to previous research [38] showing that RN leadership included both being a team leader and bearing a heavy professional responsibility, such as being accountable for the work they did not perform themselves. This study also showed RNs cooperated across care levels, which included coordinating nursing measures where several professions were involved. The next of kin experienced difficulties related to RN leadership and this cooperation where there were insufficient numbers of physicians at the healthcare centres, and the contact between the physicians and the RNs was carried out at a distance. However, the next of kin in this study believed that the cooperation difficulties were not the RNs’ fault; instead, they related these difficulties to a lack of organisational preconditions.

### Limitations

In qualitative research, the focus is on the phenomenon being explored; therefore, the number of participants is not of pertinence [21, 26]. The prevailing circumstances resulting from the ongoing coronavirus pandemic during data collection were a factor that contributed to the number of participants being 11*.* Nevertheless, the included participants were able to contribute to a rich description of the phenomenon [21]. On the one hand, the interviews were conducted by telephone and not face to face, which could be a limitation [20] that affects the credibility. On the other hand, the participants were all under the same conditions to participate, and there was no influence from the interviewer, which strengthened the dependability. The interviewer had no information about environmental factors that could have influenced participants during the interviews, which could have affected the transferability. However, all authors cooperated and were involved in the analysis and the writing of the manuscript, hence strengthening the trustworthiness of the study regarding credibility and dependability [22–24].

## Conclusions

This study contributes to important knowledge on the experiences of next of kin regarding RN leadership. This knowledge can contribute to the development of nursing education that will strengthen and prepare RNs in their important role as a leader to older adults in municipal home care. The recommendation is that the knowledge from next of kin’s experiences of RN leadership needs to be communicated as an important part of RNs’ professional role that contributes to good and safe care for older adults with municipal home care and for their next of kin in the education of RNs.

This knowledge can also be important to managers and decision makers when improving policies regarding organisational preconditions for RNs to perform their leadership. This can be done by educating managers and decision makers for municipal home care about the importance of RN leadership as a prerequisite for good and safe care for older adults with municipal home care and their next of kin. It is important to meet future challenges associated with the increasing number of older adults in need of care at home where RNs play the most important roles. Further research is needed to explore how to strengthen this leadership from more perspectives to meet future challenges.

## Data Availability

The datasets used and/or analysed during the current study are available from the corresponding author on reasonable request.

## Abbreviations

**RN**: Registered nurse
